# Supplementary material for: Resolving the full spectrum of human genome variation using Linked-Reads
Source: Genome Res. 2019 Apr;29(4):635–45. doi: 10.1101/gr.234443.118 (PMC6442396; doi:10.1101/gr.234443.118)
Supplement: Supplemental Material [file supp_gr.234443.118_Supplemental_longranger-2.2.zip › longranger-2.2.tar.bz2!/lr2.2-public/lib/stan/cmdstan/stan/doc/api/html/mcmc_2sample_8hpp_source.html]

Stan: src/stan/mcmc/sample.hpp Source File


|  |  |
| --- | --- |
|  | Stan  2.5.0  probability, sampling & optimization |


- Main Page
- Related Pages
- Namespaces
- Classes
- Files

- File List
- File Members

All Classes Namespaces Files Functions Variables Typedefs Enumerations Enumerator Friends Macros Pages

- src
- stan
- mcmc

sample.hpp

Go to the documentation of this file.

1 #ifndef STAN\_\_MCMC\_\_SAMPLE\_\_HPP

2 #define STAN\_\_MCMC\_\_SAMPLE\_\_HPP

3

4 #include <vector>

5 #include <string>

6

7 #include <stan/math/matrix/Eigen.hpp>

8

9 namespace stan {

10

11  namespace mcmc {

12

13  class sample {

14

15  public:

16

17  sample(const Eigen::VectorXd& q,

18  double log\_prob,

19  double stat) :

20  cont\_params\_(q),

21  log\_prob\_(log\_prob),

22  accept\_stat\_(stat) {};

23

24  virtual ~sample() {}; // No-op

25

26  int size\_cont() const {

27  return cont\_params\_.size();

28  }

29

30  double cont\_params(int k) const {

31  return cont\_params\_(k);

32  }

33

34  void cont\_params(Eigen::VectorXd& x) const {

35  x = cont\_params\_;

36  }

37

38  const Eigen::VectorXd& cont\_params() const {

39  return cont\_params\_;

40  }

41

42  inline double log\_prob() const {

43  return log\_prob\_;

44  }

45

46  inline double accept\_stat() const {

47  return accept\_stat\_;

48  }

49

50  void get\_sample\_param\_names(std::vector<std::string>& names) {

51  names.push\_back("lp\_\_");

52  names.push\_back("accept\_stat\_\_");

53  }

54

55  void get\_sample\_params(std::vector<double>& values) {

56  values.push\_back(log\_prob\_);

57  values.push\_back(accept\_stat\_);

58  }

59

60  private:

61

62  Eigen::VectorXd cont\_params\_; // Continuous coordinates of sample

63  double log\_prob\_; // Log probability of sample

64  double accept\_stat\_; // Acceptance statistic of transition

65

66  };

67

68  } // mcmc

69

70 } // stan

71

72 #endif

73

stan::mcmc::sample::accept\_stat

double accept\_stat() const

**Definition:** sample.hpp:46

stan::mcmc::sample::~sample

virtual ~sample()

**Definition:** sample.hpp:24

stan::mcmc::sample::size\_cont

int size\_cont() const

**Definition:** sample.hpp:26

stan::mcmc::sample::log\_prob

double log\_prob() const

**Definition:** sample.hpp:42

stan::mcmc::sample::get\_sample\_params

void get\_sample\_params(std::vector< double > &values)

**Definition:** sample.hpp:55

stan::mcmc::sample::cont\_params

const Eigen::VectorXd & cont\_params() const

**Definition:** sample.hpp:38

stan::mcmc::sample::sample

sample(const Eigen::VectorXd &q, double log\_prob, double stat)

**Definition:** sample.hpp:17

stan::mcmc::sample::cont\_params

double cont\_params(int k) const

**Definition:** sample.hpp:30

stan::mcmc::sample::cont\_params

void cont\_params(Eigen::VectorXd &x) const

**Definition:** sample.hpp:34

Eigen.hpp

stan::mcmc::sample::get\_sample\_param\_names

void get\_sample\_param\_names(std::vector< std::string > &names)

**Definition:** sample.hpp:50

stan::mcmc::sample

**Definition:** sample.hpp:13


---

[ Stan Home Page ]

*© 2011–2014,
Stan Development Team.*
